# Diversity of Integration Sites of Bovine Leukemia Virus (BLV) and Roles of Genes Related to Development of BLV-Induced Lymphoma in a Large Cohort

**DOI:** 10.3390/ijms27020727

**Published:** 2026-01-10

**Authors:** Ryosuke Matsuura, Meripet Polat Yamanaka, Noriko Fukushi, Susumu Saito, Keisuke Fukumoto, Kazuyoshi Hosomichi, Shin-nosuke Takeshima, Yoko Aida

**Affiliations:** 1 Laboratory of Global Infectious Diseases Control Science, Graduate School of Agricultural and Life Sciences, 1-1-1 Yayoi, Bunkyo-ku 113-8657, Tokyo, Japan; matsuura-ryosuke@g.ecc.u-tokyo.ac.jp (R.M.); meripet513@gmail.com (M.P.Y.); fukushi-noriko@g.ecc.u-tokyo.ac.jp (N.F.); kabe0912@t-net.ne.jp (S.S.); takesima@jumonji-u.ac.jp (S.-n.T.); 2 Support Unit for Bio-Material Analysis, Research Resources Division, RIKEN Center for Brain Science, 2-1 Hirosawa, Wako 351-0198, Saitama, Japan; keisuke.fukumoto@riken.jp; 3 Laboratory of Computational Genomics, School of Life Science, Tokyo University of Pharmacy and Life Sciences, 1432-1 Horinouchi, Hachioji 192-0392, Tokyo, Japan; khosomic@toyaku.ac.jp; 4 Department of Food and Nutrition, Faculty of Human Life, Jumonji University, 2-1-28 Sugasawa, Niiza 352-8510, Saitama, Japan

**Keywords:** bovine leukemia virus, enzootic bovine leukosis, integration site, gene ontology analysis, protein–protein interaction analysis, proviral DNA-capture-seq

## Abstract

Bovine leukemia virus (BLV) causes enzootic bovine leukosis (EBL), yet its pathogenic mechanisms remain largely unclear. In particular, the role of BLV genomic integration sites (IS(s)) in BLV-induced leukemogenesis has not been fully elucidated. Here, we identified a total of 235 ISs from 99 BLV-infected cattle with lymphoma, of which 4.3% and 46.8% were located within exon and intron, respectively, while no preferential integration into CpG islands or repetitive regions was observed. All identified ISs were distinct, and no identical sites were detected among the samples. We identified 246 genes related with these ISs and protein–protein interaction analysis of these genes demonstrated one “IS-Clustered genes” composed of 85 among 246 genes. This “IS-Clustered genes” contains 12 cancer genes (14.1%) with high significantly proportion. Notably, with 55 among 99 cattle tested (55.6%) harboring ISs within this cluster, suggesting its crucial involvement in BLV-induced pathogenesis. Furthermore, integrated analysis of known lymphoma- and IS-related genes and the 85 “IS-Clustered genes” showed that key genes formed a shared cluster, indicating a potential “common EBL-associated cluster.” These findings provide important insights into the role of BLV integration in EBL development and may contribute to elucidating its molecular mechanisms underlying onset of EBL. In addition, these findings may also aid in the development of therapeutic strategies and facilitate early diagnosis.

## 1. Introduction

Bovine leukemia virus (BLV), which is closely related to human T-cell leukemia virus type 1 (HTLV-1), is the causative agent of enzootic bovine leukosis (EBL), the most common neoplastic disease among cattle [1,2]. Approximately 70% of BLV-infected cattle are asymptomatic carriers; the remaining 30% of infected cattle show persistent lymphocytosis, which is characterized by polyclonal expression of non-neoplastic CD5-positive B lymphocytes [1,3]. Only 1–5% of cattle develop B-cell leukemia/lymphoma after a prolonged latency period [1].

EBL is a multifactorial disease; however, the mechanisms of its pathogenesis are not fully understood. As a viral factor, the proviral load of BLV is associated with disease progression [4,5]. In addition, the nucleotide at position 175 in its long terminal repeat (LTR) and the amino acid at position 233 in the Tax protein are associated with the onset of EBL [6,7]. As a host factor, the major histocompatibility complex, which plays a crucial role in immune regulation, also contributes to the onset of EBL. In particular, some bovine leukocyte antigen *DRB3* alleles confer resistance and susceptibility to proviral loads and lymphoma [8,9,10,11,12,13,14,15,16]. Wide distribution of mutations in the tumor suppressor gene *TP53* has been reported in cattle with lymphoma [17,18,19,20,21]. Additionally, somatic mutations in *TP53* as well as various other cancer-associated genes have been reported [22]. Furthermore, changes in the expression levels of certain genes, such as Protein Arginine Methyltransferase 5 (*PRMT5*) and those associated with DNA Mismatch repair, have been reported [23,24].

Similar to other retroviruses, the BLV genome integrates into the host genome as a provirus and induces lifelong infections [1]. Many studies have investigated the association between BLV integration sites (IS(s)) and the onset of EBL [25,26,27,28,29,30,31,32]. BLV proviruses are distributed across every chromosome, and there are no integration hotspots [25]. Indeed, some studies have shown that the BLV provirus does not favor integration near transcription start sites, CpG islands, or repetitive sequences such as transposons [25,26]. In contrast, it is reported that BLV proviruses were integrated near CpG islands and into long interspersed nuclear elements (LINE) more frequently in cattle with lymphoma under 3 years old than in those over 3 years old [28]. Therefore, the impact of BLV integration into specific regions of the genome on the development of EBL remains unclear.

Furthermore, integration of the BLV genome into exons or introns is presumed to exert a substantial influence on gene expression. In addition, proviruses can disrupt the mRNA expression of host genes located in their vicinity through gene interruption [27]. Indeed, BLV/HTLV-1 proviruses in the tumor genomes of lymphoma animals or adult T-cell leukemia/lymphoma patients are preferentially integrated near cancer driver genes [27,33,34,35,36]. Additionally, clone abundance positively correlates with the proximity of the provirus to the transcribed region [26,28]. Notably, integration of the BLV genome has been identified near the genes that play a role in cancer development, such as in the intergenic region of *RTN4IPI* and *ATG5*, and in the intron of the regulatory-associated protein of mTOR (*RPTOR*) in two B-cell lymphoma lines [31]. Additionally, integration events within an intron of the checkpoint kinase 2 (*CHEK2*) gene and in the intergenic region of clarin 1 (*CLRN1*) and siah E3 ubiquitin protein ligase 2 (*SIAH2*) were observed at the onset of lymphoma in naturally infected BLV individuals who progressed from a premalignant stage to terminal disease [32]. Nevertheless, the interrelationships between these genes remain unclear, and the specific contributions of individual genes to disease onset are yet to be elucidated. Therefore, although ISs are believed to contribute to the onset of EBL, the mechanisms underlying their involvement in disease pathogenesis remain largely unresolved.

To elucidate the mechanisms underlying the contribution of ISs to the development of EBL, we previously developed a proviral DNA capture sequencing (proviral DNA-capture-seq) method [31], involving a target-enrichment high-throughput sequencing system for the characterization of BLV ISs. In this study, we firstly collected samples from 99 naturally BLV-infected cattle with lymphoma nationwide and identified ISs. Secondly, 235 ISs identified across 99 EBL cattle revealed that the BLV genome exhibited a preference for integration into exon and intron regions but not into repetitive regions. Thirdly, we successfully extracted 246 IS-Related genes and their functional annotations using Gene Ontology (GO) analysis. Fourth, through protein–protein interaction network analysis, we detected one cluster comprising 85 genes, and 55 of the 99 cattle with lymphoma possessed one or more ISs associated with these clustered genes. Finally, an integrated analysis of these 85 clustered genes using previously reported IS- and EBL-related genes was performed and one “common EBL-related cluster” comprising 132 genes was found and this “common EBL-related cluster” was strongly associated with cancer pathways. Because monoclonal expansion has been observed prior to the onset of EBL, and ISs are maintained from the premalignant stage through terminal disease [32], our results demonstrate that this “common EBL-related cluster” may be useful for the early detection of the onset of EBL.

## 2. Results

### 2.1. Distribution Analysis Revealed That BLV Proviruses Exhibit a Preference for Integration into Exon and Intron

**Figure 1:**
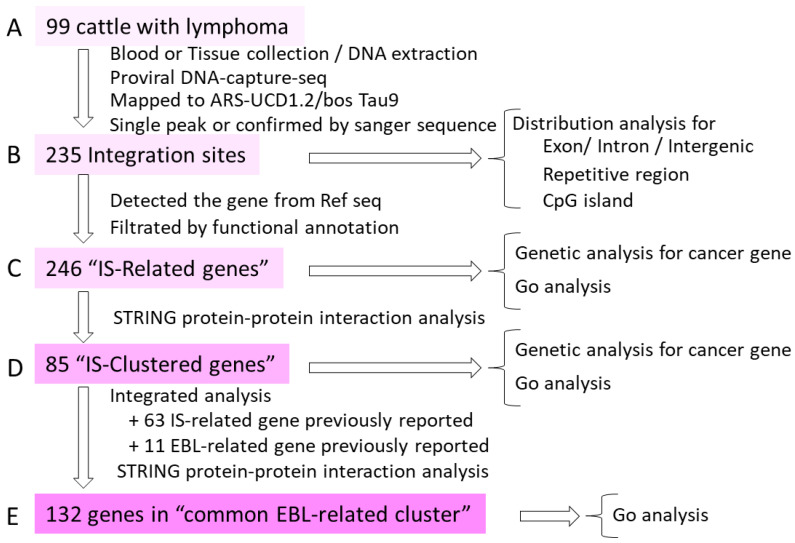
Scheme for integration site analysis of 99 EBL cattle.

To elucidate the contribution of BLV ISs to the development of EBL, DNA was extracted from blood or tissue samples obtained from 99 cattle with lymphoma collected in a nationwide survey across Japan (Figure 1A). Distribution of the BLV provirus was screened using the proviral DNA-capture-seq method (Figure 1A). Sequencing reads were mapped to the bovine reference genome (ARS-UCD1.2/bos Tau9) (Figure 1A). Genomic regions displaying mapped reads exhibiting a single peak or regions confirmed to contain chimeric sequences of BLV and host DNA through PCR and Sanger sequencing were defined as ISs (Figure 1A).

**Figure 2:**
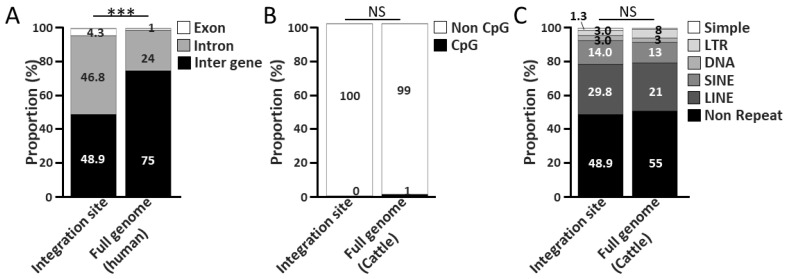
BLV proviral integration sites and their distribution in the bovine genome. (**A**) The columns represent the proportion of BLV proviral integration sites for the exon, introns, and inter-genes, respectively. Proportions of the exons, introns, and inter-genes in the human genome were used as the references [33]. (**B**) The black and white columns represent the proportion of BLV proviral integration sites for the CpG island and non-CpG island regions, respectively. Proportions of the CpG island of the cattle genome were used for the reference. (**C**) The columns represent the proportions of BLV proviral integration sites for the repetitive regions, simples, LTRs, DNAs, SINEs, and LINEs, and non-repeat regions, respectively. Repetitive regions of BLV genomes identified by repeat maskers was used for the reference. Asterisks indicate statistical differences (*** *p* < 0.001). ‘NS’ indicates no statistical difference.

Two-hundred and thirty-five ISs were identified from the samples of 99 cattle with lymphoma, with the number of ISs per animal ranging from 1 to 12 (mean, 2.4) (Figure 1B). In particular, all the identified ISs were distinct, and no shared sites were detected among the samples. To elucidate the distribution of ISs within the bovine genome, ISs were categorized into exons, introns, and intergenic regions. Among 235 ISs, 10 (4.3%) were mapped to exons, 110 (46.8%) to introns, and 115 (48.9%) to inter-genes (Figure 2A). Exons, introns, and intergenic regions constitute approximately 1%, 24%, and 75% of the human genome [36], respectively. The ISs identified in EBL cattle were significantly enriched in exons and introns compared with those in the human genome (*p* < 0.001). Human genomic data were used for this analysis because corresponding bovine genomic data were not available. Using human gene composition as a reference for mammals is justified because less than 1% of genes lack detectable orthologs between humans and mice [37]. The proportion of ISs located within repetitive regions was analyzed. Among the 235 ISs, none were located within CpG islands, which comprise approximately 1% of the bovine genome (Figure 2B). This observation is consistent with the expected theoretical distribution. In addition, among the 235 ISs, 3 (1.3%) were mapped to simple repeats, 7 (3.0%) to LTR, 7 (3.0%) to DNA repeat elements (DNA), 33 (14.0%) to short interspersed nuclear elements (SINEs), 70 (29.8%) to LINE, and 115 (48.9%) to non-repeat regions (Figure 2C). Simple repeats, LTRs, DNA, SINEs, LINEs, and non-repeat regions constitute approximately 0%, 8%, 3%, 13%, 21%, and 55% of the cattle genome identified by repeat maskers, respectively. The proportion of ISs within repetitive regions appeared to be consistent with the expected theoretical value. These results suggest that ISs tend to be inserted near genes but lack a preference for CpG islands and repetitive regions.

### 2.2. Extraction of “IS-Related Genes” and Genetic Analysis Revealed That the Effects of BLV Integration near Cancer-Related Genes on the Onset of EBL Were Limited

**Figure 3:**
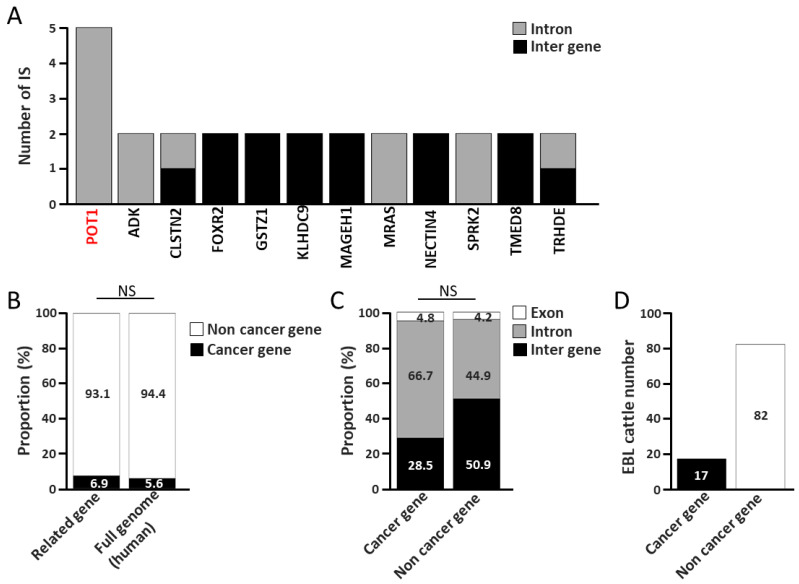
Analysis of “IS-Related genes”. (**A**) The columns represent the number of “IS-Related genes” associated with multiple integration sites. Red text indicates cancer genes. (**B**) The columns represent the proportion of “related genes” for cancer-related genes and non-cancer-related genes classified using the OncoKB^TM^ Cancer Gene List. Proportions of the cancer gene of human genome was used for the reference. (**C**) The columns represent the proportion of cancer-related genes and non-cancer related genes associated with BLV proviral integration sites for the exons, introns, and inter-genes, respectively. (**D**) The columns represent the number of EBL cattle possessing one or more ISs associated with cancer-related genes and those of the others. NS indicates no statistical difference.

To examine the potential direct role of ISs of BLV in tumorigenesis, genes harboring BLV provirus insertions within their exons or introns and the closest upstream and downstream genes to each IS were designated “IS-Related genes” (Figure 1B). From a total of 350 genes (including 10 ISs located in exons, 110 ISs located in introns, and 115 ISs located in intergenic regions), the 246 genes with functional annotations were extracted using the Database for Annotation, Visualization, and Integrated Discovery (DAVID) and defined as “IS-Related genes” (Table 1) (Figure 1B,C). These 246 genes were classified into groups containing 8, 95, and 145 genes related to IS, located in exons, introns, and intergenic regions, respectively. Interestingly, only 12 genes (genes with five ISs, *PUT1*; genes with two ISs, *ADK*, *CLSTN2*, *POXR2*, *GSTZ1*, *KLHDC9*, *MAGEH1*, *MRAS*, *NECTIN4*, *SPRK2*, *TMED8*, and *TRHDE*) were associated with multiple IS (Table 1, shown in the boxes, and Figure 3A).

Comparison between the reference genes in the OncoKBTM Cancer Gene List (https://www.oncokb.org/cancer-genes, accessed on 8 October 2025) and the 246 “IS-Related genes” clearly showed that only 17 genes (6.9%), namely *IL10*, *BPHB1*, *ERBB4*, *HDAC4*, *ITK*, *MSH3*, *POT1*, *PRDM16*, *PTEN*, *SGK1*, *SMARCA4*, *TPR*, *CHN1*, *DPYD*, *LRRK2*, *MART1*, and *TBL1XR1*, as shown in red in Table 1 among the 246 “IS-Related genes” cancer genes (Figure 3B and Table 1, shown in red). Notably, five ISs were detected within the introns of *POT1*, a major cancer gene. In contrast, among the 21,306 known human genes [38], 1203 are classified as cancer genes according to the OncoKBTM Cancer Gene List, representing approximately 5.6% of all genes. Thus, the proportion of cancer genes among the “IS-Related genes” was 6.9% (Figure 3B and Table 1, shown in red), suggesting no significant deviation from the expected theoretical value. In addition, the distribution of IS located in exons, introns, and intergenic regions was examined among the cancer-related and non-cancer genes, revealing no significant differences between the two groups (Figure 3C). Furthermore, among 99 cattle with lymphoma, only 17 (17.2%) possessed one or more ISs associated with cancer-related genes (Figure 3D). These results demonstrate that the influence of ISs located near cancer-related gene on BLV-induced leukemogenesis is limited.

### 2.3. GO Analysis of “IS-Related Gene” Revealed That a Subset of Related Genes Is Involved in Pathways for Oncogenesis

GO analysis was conducted to clarify the mechanisms of BLV-induced leukemogenesis from the functional perspective of “IS-Related genes” (Figure 1C). As shown in Table 2 and Table 3, 21 GO terms associated with biological processes and 6 KEGG pathways were identified (Table 2 and Table 3). In particular, GO terms related to biological processes potentially involved in tumorigenesis, such as “positive regulation of receptor signaling pathway via JAK-STAT,” “protein polyubiquitination,” “protein dephosphorylation,” “positive regulation of transcription by RNA polymerase II,” and “cell adhesion,” were observed (Table 2, shown in underlined). These GO terms are related to signal pathways and transcription. These findings suggest that integration of the BLV provirus may disturb these biological processes, thereby contributing to lymphoma development. However, no KEGG pathways associated with cancer were identified (Table 3). Thus, 38 “IS-Related genes” (*CAMK2A*, *ERBB4*, *GHR*, *IL10*, *FBXL17*, *FBXO4*, *HECTD2*, *DTL*, *RNF144A*, *UBE3D*, *RNGTT*, *DUSP10*, *PTEN*, *PPM1A*, *PTPRM*, *LDB2*, *POU3F3*, *SMARCA4*, *TBL1XR1*, *TEAD1*, *TEF*, *CCDC32*, *HDAC4*, *HOXA2*, *RFX7*, *SLC30A9*, *TOX*, *TFAP2A*, *ZNF407*, *EPHB1*, *FER*, *ACAN*, *CNTNAP2*, *NECTIN4*, *PARVA*, *PCDH7*, *TINAG*, and *VCL*) were associated with these five GO terms, and only 6 genes, such as *ERBB4*, *IL10*, *SMARCA4*, *TBL1XR1*, *HDAC4* and *EPHB1* were cancer genes. These results suggest that non-oncogenes, as well as oncogenes, may be involved in the onset of EBL. However, this finding is unlikely to entirely facilitate the development of lymphoma, as only 6 cancer genes and 32 non-cancer genes were associated with five GO terms (Table 2, shown in underlined).

### 2.4. Analysis of “IS-Clustered Genes” Revealed That the Clustered Genes Were Strongly Associated with the Onset of EBL

**Figure 4:**
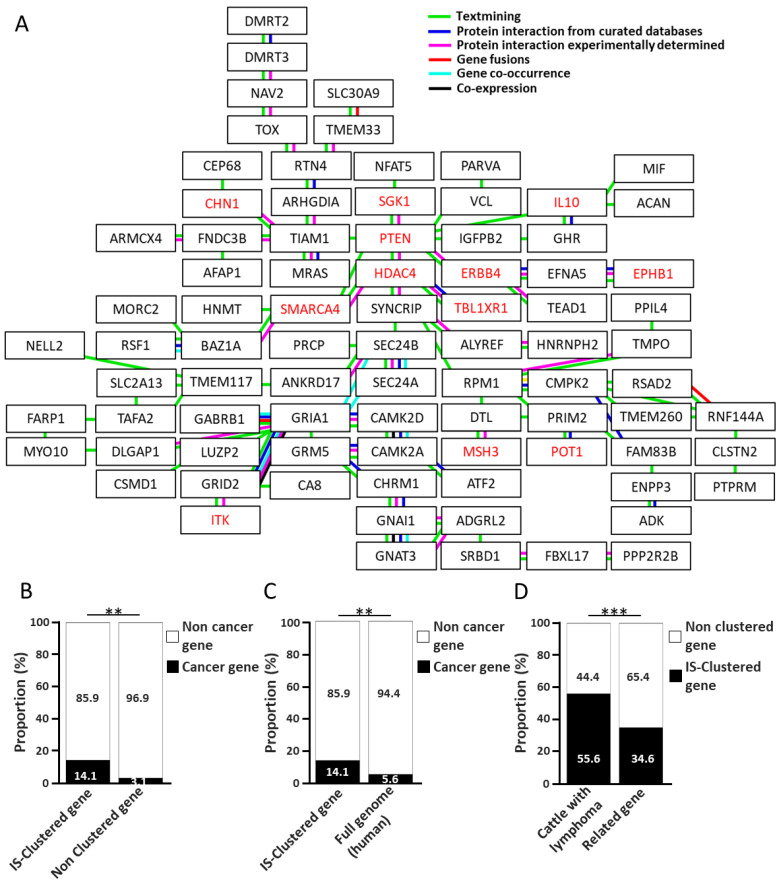
Protein–protein interaction network of “IS-Clustered genes”. (**A**) Columns represent each gene, and lines represent protein–protein interactions. Protein–protein interactions are highlighted as follows: Green indicates “Textmining”; blue indicates “Protein interaction from curated databases”; pink indicates “Protein interaction experimentally determined”; red indicates “Gene fusions”; light blue indicates “Gene co-occurrence”; and black indicates “Co-expression”. (**B**) The columns represent the proportion of IS-clustered genes and non-clustered genes for cancer-related gene and non-cancer genes, respectively. Proportions of the cancer genes within the human genome were used as the reference. (**C**) The columns represent the proportion of IS-clustered cancer-related and non-cancer-related genes, respectively. Proportions of the cancer-related genes within the human genome were used as the reference. (**D**) The columns represent the number of EBL cattle possessing one or more ISs associated with “IS-Clustered genes” and others. Significance was analyzed using Fisher’s test. Asterisks indicate statistical differences (** *p* < 0.01, *** *p* < 0.001).

Not all ISs are implicated in lymphoma; therefore, to identify genes involved in oncogenic processes, the “IS-Related genes” were clustered using protein–protein interaction networks using STRING (https://string-db.org/, accessed on 8 October 2025) (Figure 1D). Remarkably, of the 246 “IS-Related genes”, 85 genes (Table 1, shown in bold) were found to form a single cluster within the protein–protein interaction network (Figure 4A). These 85 genes were defined as “IS-Clustered genes”. In addition, these “IS-Clustered genes” included 12 cancer genes (14.1%), namely *CHN1*, *SGK1*, *IL10*, *PTEN*, *HDAC4*, *ERBB4*, *BPHB1*, *SMARCA4*, *TBL1XR1*, *ITK*, *MSH3* and *POT1*, as shown in red color in Figure 4. In addition, the proportion of cancer genes of these clustered genes was significantly (*p* < 0.01) higher than that of the 161 non-clustered genes (3.1%) (Figure 4B). Furthermore, this proportion was also significantly (*p* < 0.01) higher than the proportion of cancer genes in the human genome (5.6%) (Figure 4C). These results suggest that the “IS-Clustered genes” play an important role in lymphoma development. Indeed, although 84 (34.6%) of the 246 “IS-Related genes” were classified as “IS-Clustered genes,” 55 (55.6%) of the 99 cattle with lymphoma possessed one or more ISs associated with the “IS-Clustered genes,” and the proportion was significantly (*p* < 0.001) higher than that of the non-clustered genes (Figure 4D).

### 2.5. GO Analysis Revealed That the “IS-Clustered Genes” Have a Role in the Onset of EBL

To investigate the functional roles of the “IS-Clustered genes,” GO analysis was conducted. As shown in Table 4 and Table 5, 13 GO terms associated with biological processes and 17 KEGG pathways were identified. Compared with “IS-Related genes,” the number of GO terms associated with biological processes decreased in “IS-Clustered genes,” whereas the number of KEGG pathways increased. These results suggest that “IS-Clustered genes” were enriched for gene groups that function more cooperatively within specific biological pathways. In particular, GO terms related to biological processes involved in tumorigenesis, such as “positive regulation of receptor signaling pathway via JAK-STAT,” “signal transduction,” “positive regulation of miRNA transcription,” “NK T cell differentiation,” “protein phosphorylation,” and “positive regulation of toll-like receptor 9 signaling pathway” (Table 4, shown in underlined), and KEGG pathways involved in tumorigenesis, such as “PI3K-Akt signaling pathway” and “Proteoglycans in cancer” (Table 5, shown in underlined) were detected. In addition, these GO terms and KEGG pathways were related to signal transduction, transcriptional regulation, and immune regulation, leading to the regulation of cancer. Notably, “NK T cell differentiation” was detected, consistent with previous reports showing that BLV reduces antiviral cytokine activities and NK cell cytotoxicity by inducing TGF-β secretion from regulatory T cells [39]. Furthermore, theses 6 GO terms and 2 KEGG pathways contained a total of 26 (30.6%) genes (*CAMK2A*, *ERBB4*, *GHR*, *IL10*, *ADGRL2*, *CAMK2D*, *CHN1*, *DUSP10*, *FAM83B*, *GABRB1*, *MYO10*, *PTPRM*, *SMARCA4*, *TEAD1*, *ITK*, *ATF2*, *EPHB1*, *ITK*, *SGK1*, *RSAD2*, *RTN4*, *CHRM1*, *EFNA5*, *PTEN*, *PPP2R2B*, *SGK1*, *TIAM1*, and *MRAS*) among the 85 “IS-Clusterd genes.” In addition, these 26 genes included eight cancer genes, namely *CHN1*, *EPHB1*, *ERB4*, *IL10*, *ITK*, *PTEN*, *SGK1*, and *SMARCA4* (Table 4 and Table 5, shown in red). These results suggest that the clustered genes possess multiple physiological functions that are critically important for the onset of EBL.

### 2.6. Discovery of “Common EBL-Related Clusters” Through Integrated Analysis of the “IS-Clustered Genes” and Previously Reported Genes

**Figure 5:**
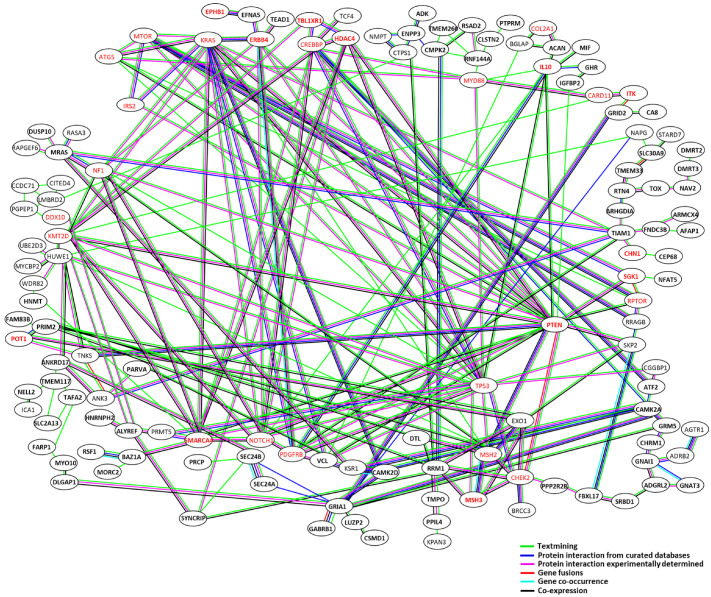
Protein–protein interaction network of “common EBL-related clusters”. Columns represent each gene, and lines represent protein–protein interactions. Red text indicates cancer genes and bold text indicates “IS-Clustered genes”. Protein–protein interactions are indicated as follows: Green indicates “Textmining”; Blue indicates “Protein interaction from curated databases”; Pink indicates “Protein interaction experimentally determined”; Red indicates “Gene fusions”; Light blue indicates “Gene co-occurence”; and Black indicates “Co-expression”.

Numerous ISs have been identified, and 65 genes associated with these IS have been reported (Table 6). However, only *FOXR2* was identified in the present study. This finding suggests that there are no apparent hotspots of BLV proviral integration. Furthermore, several BLV-induced lymphoma-related genes, including those harboring somatic mutations or showing altered expression levels in cattle with EBL, have been previously reported (Table 7). Among these, only *PTEN* was identified in the present study. To elucidate the relationship between the “IS-Clustered genes” and previously reported genes, protein–protein interactions were analyzed using STRING (Figure 1E). Consequently, 49 of the 75 previously reported genes were incorporated into a single cluster (Table 6 and Table 7, shown in bold) and 132 genes were included in this cluster (Figure 5). Notably, all the genes listed in Table 7 were incorporated into this cluster (Figure 5 and Table 7). These results suggest that the cluster identified in the present study may play a central role in the onset of EBL. Therefore, we defined this cluster as “common EBL-related cluster.”

### 2.7. GO Analysis Revealed That “Common EBL-Related Clusters” Have Various Functions in the Onset of EBL

To investigate the functional roles of the “common EBL-related cluster,” GO analysis was conducted (Figure 1E). As shown in Table 8 and Table 9, 43 GO terms associated with biological processes and 49 KEGG pathways were identified. Ten GO terms, namely “positive regulation of receptor signaling pathway via JAK-STAT,” “modulation of chemical synaptic transmission,” “signal transduction,” “nervous system development,” “ephrin receptor signaling pathway,” “positive regulation of synapse assembly,” “positive regulation of miRNA transcription,” “NK T cell differentiation,” “protein phosphorylation,” and “positive regulation of toll-like receptor 9 signaling pathway” were identified in the same “IS-Clustered genes” and 33 GO terms were newly detected. Similarly, 14 KEGG pathways, including “Dopaminergic synapse,” “PI3K-Akt signaling pathway,” “Amphetamine addiction,” “Long-term potentiation,” “Adrenergic signaling in cardiomyocytes,” “cAMP signaling pathway,” “Nucleotide metabolism,” “Axon guidance,” “Proteoglycans in cancer,” “Cholinergic synapse,” “Pyrimidine metabolism,” “Long-term depression,” “Calcium signaling pathway,” and “Cushing syndrome were identified in the same “IS-Clustered genes” and 35 KEGG pathways were newly detected.

In particular, several KEGG pathways involved in cancer were detected. Briefly, seven KEGG pathways were detected, namely “MicroRNAs in cancer,” “Pathways in cancer,” “prostate cancer,” “Colorectal cancer,” “Central carbon metabolism in cancer,” “Breast cancer,” and “PD-L1 expression and PD-1 checkpoint pathway in cancer”, which are directly involved in cancer (Table 9, shown in underlined). In addition, “Proteoglycans in cancer” was shared across “IS-Clustered genes.” These eight pathways comprised 23 genes (*CREBBP*, *KRAS*, *EFNA5*, *HDAC4*, *IRS2*, *MTOR*, *NOTCH1*, *PTEN*, *PDGFRB*, *RPTOR*, *TP53*, *TIAM1*, *ANK3*, *CAMK2A*, *CAMK2D*, *ERBB4*, *MRAS*, *GNAI1*, *SKP2*, *AGTR1*, *MSH2*, *MSH3*, *MYD88*) and 10 genes (*EFNA5*, *HDAC4*, *PTEN*, *TIAM1*, *CAMK2A*, *CAMK2D*, *ERBB4*, *MRAS*, *GNAI1*, and *MSH3*), which were “IS-Clustered genes” (Table 9, shown in bold). Furthermore, 14 cancer-related genes were identified (*CREBBP*, *KRAS*, *HDAC4*, *IRS2*, *MTOR*, *NOTCH1*, *PTEN*, *PDGFRB*, *RPTOR*, *TP53*, *ERBB4*, *MSH2*, *MSH3*, and *MYD88*) among these 23 genes (Table 9, shown in red). These results suggest that the “common EBL-related cluster” possesses multiple functions that are critically important in the onset of EBL, and integration of the BLV genome into the nearby “common EBL-related cluster” may lead to the onset of EBL.

## 3. Discussion

In this study, a novel analysis of ISs in a cohort of 99 cattle with lymphoma was conducted. First, we detected 235 ISs from 99 BLV-infected cattle with lymphoma, and identified 246 associated genes as “IS-Related genes.” Next, we extracted a single cluster within the 246 genes, comprising 85 genes identified as “IS-Clustered genes.” Finally, a “common EBL-related cluster” including 132 genes was discovered through integrated analysis of previously reported EBL- and IS-related genes within the “IS-Clustered genes.” This study is the first to elucidate the mechanism of EBL onset through protein–protein interaction analysis of IS-Related genes. Notably, ISs associated with the cluster comprising 85 genes, including 12 oncogenes, were conserved in 55 of the 99 BLV-infected cattle with lymphoma, suggesting a potential role of IS in the onset of EBL. These findings offer significant insights into the relationship between BLV integration and EBL pathogenesis, which remains poorly understood.

In the present study, we found the “common EBL-related cluster” through the integration analysis of “IS-Clustered genes” and previously reported integration side-associated genes and EBL-related genes. This cluster included *TP53*, which has been reported to be associated with EBL development in numerous studies [17,18,19,20,21]. Additionally, in this study, *TP53*, which is similar to other cancer genes such as *PTEN* and *SMARCA4*, exhibits many interactions with other genes within the network of the “common EBL-related cluster.” Moreover, this cluster appears to represent a potential serve as a missing link between several genes that we previously reported as EBL-related genes, including *PRMT5*, *MSH2*, *EXO1*, *RTN4IP1*, *ATG5*, *RPTOR*, and *CHEK2* [23,24,31,32]. Therefore, it is possible that integration of the BLV genome into cancer genes or genes that interact with cancer genes in the “common EBL-related cluster,” changes their expression and subsequently contributes to the onset of EBL.

In contrast, the 161 genes that were not included in “IS-Clustered genes” did not form clear clusters, and only five of them were cancer genes. This result suggests that integration sites associated with cancer-related genes may contribute to the onset of EBL, whereas other integration sites are less likely to be involved in the onset of EBL. Because factors of the onset of EBL other than integration sites, such as viral factors and host factors, are also important, further detailed analyses are required.

Consistent with the findings of previous studies, a high frequency of BLV genome insertions was observed within exons and introns in the present study [25,26]. In contrast, no preferential integration of the BLV genome into CpG islands or repetitive regions, which are considered to affect gene expression, was detected [25,26]. However, no significant integration was observed near cancer genes. This result conflicts with those of previous reports [27,33,34,35,36]. This is likely because the present study analyzed an unprecedented number of 99 cattle with lymphoma, and not all cattle developed lymphoma because of their ISs. However, cancer genes were enriched within the “IS-cluster genes” and integrated analysis detected the “common EBL-related cluster”, suggesting that the integration of the BLV genome near the “common EBL-related cluster” may be a factor in EBL onset.

In this study, a large number of “IS-Related genes” were identified. However, it remains unclear whether the expression of these genes is altered by BLV genome integration. Further studies are needed to investigate whether BLV integration directly affects the expression of these genes. Especially, to clarify the changes in RNA expression associated with ISs, it is necessary to perform RNA-seq on cattle with and without lymphoma and identify the genes that are differentially expressed between the two groups. Furthermore, integrative analyses combining RNA-seq data with the present IS data will be essential to clarify whether the “IS-Related genes” and “IS-Clustered genes” are truly involved in disease development.

Moreover, we identified a wide range of associated GO terms and KEGG pathways. In particular, the “common EBL-related cluster” was associated with KEGG pathways directly involved in tumorigenesis, such as “MicroRNAs in cancer,” “Pathways in cancer,” “prostate cancer,” “Central carbon metabolism in cancer,” “Colorectal cancer,” “Breast cancer,” “Proteoglycans in cancer,” and “PD-L1 expression and PD-1 checkpoint pathway in cancer.” However, the pathways that are critical for the development of lymphoma remain unknown. Although the onset of EBL is a multifactorial process involving not only host factors but also viral and environmental factors, exploring the pathways implicated in lymphoma development through the analysis of ISs is considered highly important for elucidating the mechanisms of EBL pathogenesis.

Furthermore, no studies have analyzed the impact of BLV viral strain variability on the distribution of ISs. Similarly, investigations assessing ISs based on the breed or geographic origin of cattle that developed EBL have not been conducted. This study also only utilized Japanese cattle; thus, we were unable to analyze whether ISs differed based on viral strain, cattle breed, or origin. Accordingly, future studies are needed to investigate whether the “common-EBL-related cluster” identified in this study represents a concept that can be applied worldwide.

In recent years, monoclonal expansion has been observed prior to the onset of EBL [32,40]. This finding suggests that the critical BLV genome integration for the onset of EBL occurs prior to lymphoma development. Therefore, monitoring BLV integration into the “common EBL-related cluster” identified in this study may enable the early detection of cattle at risk of developing EBL before clinical onset.

## 4. Materials and Methods

### 4.1. Sample Collection and Extraction of Genomic DNA

Blood and tissue samples were randomly collected from 99 BLV-infected cattle diagnosed with EBL in a nationwide survey conducted in Japan. Lymphoma was diagnosed based on both gross and histological observations and by detecting atypical mononuclear cells in the slaughterhouse. All samples were handled by veterinarians and researchers in strict accordance with good animal practice. The Animal Experiments Committee of the University of Tokyo approved this study (Approval Number: p22-2-030, accepted on 27 May 2022).

Genomic DNA was extracted from ethylenediaminetetraacetic acid (EDTA)-treated blood and tissue samples using the Wizard Genomic DNA Purification Kit (Promega Corporation, Madison, WI, USA). The extracted DNA was stored at −20 °C until further use. DNA from a bovine lymphosarcoma cell line (BSLC-KU1) and naturally BLV-infected lymphoma cattle samples (Kas-3) were used as positive controls because they carry known BLV ISs, as reported in previous studies [31,32]. DNA from Madin-Darby Bovine Kidney (MDBK) was used as a negative control.

### 4.2. NGS DNA Library Preparation and Library Enhancement by Viral-DNA Capture

DNA libraries were prepared using the KAPA Hyper Plus kit (Roche, Basel, Switzerland) according to the manufacturer’s instructions. Briefly, genomic DNA (1 μg) was enzymatically fragmented to an average length of approximately 500–600 bp, followed by end repair and addition of adenosine at the 3′-ends of the fragmented DNA. Then, index adaptors (Roche Sequencing Solutions, Inc., Santa Clara, CA, USA) were ligated into the DNA 6 fragments of each sample. Adaptor-tagged library fragments were purified using Agencourt AMPure XP beads (Beckman Coulter, Inc., Brea, CA, USA) on an NGS Magna Stand Ch YS-Model (Nippon Genetics Co., Ltd., Tokyo, Japan) according to the manufacturer’s instructions. Double-sided size selection was performed to keep fragments of 250 to 450 bp in length, using Agencourt AMPure XP beads to remove unwanted fragment sizes and low-molecular-weight materials. The adaptor-tagged purified libraries were subjected to PCR amplification and post-PCR purification. 21-test samples plus DNA from two positive controls (BLSC-KU1 & Kas-3) and a negative control (MDBK) were mixed to construct an NGS DNA library with a final concentration of 1 μg total DNA. For library enrichment, we used the Viral-DNA capture library enhancement method, which was previously established in our laboratory [31] with small modifications. Three custom-designed biotinylated probes (100 bp in length, targeting LTR) (Thermo Fisher Scientific, Waltham, MA, USA) from our previous study were used to capture virus–host chimeric DNA fragments and BLV proviral genome-only fragments to enrich the DNA library. Detailed information on biotinylated probes has been described in our previous publications [31,32]. DNA library enrichment was performed using the SeqCap EZ Hybridization and a Wash Kit (Roche NimbleGen, Pleasanton, CA, USA) following the manufacturer’s instructions for DNA probe hybridization and target capture. After the recommended washing steps were performed, the captured DNA was amplified using PCR and purified using Agencourt AMPure XP beads. DNA libraries enriched for proviral sequences were quantified using qPCR with Illumina P5 and P7 primers before sequencing. The quality and quantity of the amplified libraries were assessed using an Agilent 2100 Bioanalyzer (Agilent Technologies, Waldbronn, Germany) and a DNA 1000 Assay.

### 4.3. High-Throughput Sequencing Data Analysis

Multiplexed enriched libraries were subjected to cluster generation using the MiSeq Reagent Kit v3 (600 cycles) (Illumina Inc., San Diego, CA, USA). Raw sequences were generated as Fastq using Illumina MiSeq with 600 cycles of paired-end reads and validated by evaluating the distribution of quality scores. Validated FASTQ files were aligned using the Burrows-Wheeler Aligner tool (BWA v. 0.7.8-r455) [41] against a BLV reference sequence (accession number: EF600696), with or without the cattle reference genome (ARS-UCD1.2/bos Tau9) (ARS-UCD1.2–bosTau9–Genome–Assembly-NCBI (nih.gov)), using the BWA-MEM algorithm31. The resulting alignment map (SAM) format output is suitable for analysis using SAMTools (version1.15) [42]. Sequence quality, depth of coverage, and short-read alignment were analyzed using SAMTools [42]. PCR duplicates were removed using Picard (http://broadinstitute.github.io/picard/, accessed on 18 September 2025). The mapped data were visualized using Integrative Genomics Viewer software (IGV) (version2.12.3) [43]. To calculate the error rate of the sequencing step, we individually analyzed the sequencing quality score (Q-score) of each base read.

### 4.4. Confirmation of Provirus ISs Using Sanger Sequencing

To confirm the BLV ISs, the BLV-host genome junction was amplified using 148 PrimeSTAR GXL DNA Polymerase (Takara Bio Inc. Kusatsu, Japan). PCR was performed using two primers: one targeting the host genome and the other targeting the BLV region. Our previous publications [31,32] provide a detailed description of the PCR conditions used. The amplified PCR products were sequenced on an ABI3730xl DNA Analyzer with the same PCR primers, using an ABI PRISM Big Dye Terminator v. 3.1 Ready Reaction Cycle Sequencing Kit (Thermo Fisher Scientific, Waltham, MA USA). The obtained sequences were blasted against BLV 8 reference sequences (FLK-BLV (accession number: EF600696)) and the cattle genome (ARS-UCD1.2/bos Tau9) using NCBI BLAST (https://blast.ncbi.nlm.nih.gov/Blast.cgi, accessed on 20 September 2025) and the Genome Browser (https://genome.ucsc.edu/, accessed on 20 September 2025).

### 4.5. Gene Ontology Analysis

Gene ontology terms were identified using the DAVID (version 2021).

### 4.6. Protein Interaction Analysis

Protein–protein interactions were identified using STRING (version 12).

### 4.7. Statistical Analysis

Data were analyzed using Fisher’s exact test. A *p*-value < 0.05 was considered statistically significant. All calculations were performed using R software (version 4.3.0; R Foundation for Statistical Computing, Vienna, Austria).

## 5. Conclusions

Our study elucidated the role of IS in the development of EBL and identified a “common EBL-related cluster,” which plays a particularly important role in the onset of EBL. These findings provide a foundation for future investigations into the detailed molecular mechanisms underlying EBL and may contribute to the development of therapeutic strategies and early diagnostic tools for EBL.

## Figures and Tables

**Table 1:** 246 “IS-Related Gene” List ^1^.

**(1) Exon**									
*ASB11*	*ATXN3*	*C25H16orf96*	***ENPP3***	***GABRB1***	***IL10***	*LPO*	*MEFV*		
**(2) Intron**									
***ADK *** **^2^**	***AFAP1***	*AHCYL2*	***ANKRD17***	***CAMK2A***	***CAMK2D***	*CARTPT*	*CCDC178*	***CLSTN2 *** **^2,3^**	*DNASE1L2*
*DPYSL3*	*DPYSL5*	*DYSF*	*E2F5*	*ELOVL6*	***EPHB1***	***ERBB4***	*ETFDH*	*EXOC4*	*FAM151B*
***FARP1***	*FER*	***FNDC3B***	*GFPT1*	*GON4L*	*GPM6B*	***GRID2***	***GRM5***	***HDAC4***	*HIBADH*
*IBTK*	*IP6K3*	***ITK***	*KCNQ5*	*KUF27*	*LDB2*	*LOC789384*	*LRP2*	*MAST2*	*MUCR1*
*MFSD12*	*MKLN1*	***MORC2***	***MRAS *** **^2^**	*MROH2B*	*MRPL18*	***MSH3***	*MYO1E*	***MYO10***	***NAV2***
*NAV3*	***NFAT5***	*NFYC*	*NIDT9*	*OSBPL3*	*OUTD7A*	*PAFAH2*	*PEBP4*	***POT1 *** **^2^**	*PPP1R1C*
*PPP2R2B*	*PRCP*	*PRDM16*	***PTEN***	***PTPRM***	*PYROXD1*	***RNF144A***	*RNGTT*	***RRM1***	***RSF1***
***RTN4***	*SBSPON*	***SEC24A***	***SEC24B***	***SGK1***	*SHLD1*	*SLC25A14*	***SMARCA4***	*SNED1*	***SRBD1***
*SRPK2 * ^2^	*SYN3*	***TAFA2***	*TBX19*	*TCERG1L*	*TEX14*	***TIAM1***	*TMEM178B*	***TMEM260***	*TPR*
*TRHDE * ^2,3^	*TSPAN17*	*UBE3D*	***VCL***	*ZNF407*					
**(** **3) Intergenic**									
***ACAN***	***ADGRL2***	*AKAIN1*	*ALKAL2*	*ALPK2*	***ALYREF***	*APIP*	***ARHGDIA***	*ARHGEF10*	*ARL14*
*ARMC1*	***ARMCX4***	***ATF2***	*ATXN7L3B*	***BAZ1***	*C10H14orf39*	*C23H6orf141*	*C5AR1*	*C5AR2*	***CA8***
*CCDC57*	*CCDC62*	*CDH7*	*CDH10*	*CDH19*	*CDK5RAP2*	***CEP68***	***CHN1***	***CHRM1***	***CLSTN2 *** **^2,3^**
***CMPK2***	*CNTNAP2*	*COL22A1*	***CSMD1***	*CYLC1*	*CYSTM1*	*DLGAP1*	***DMRT2***	***DMRT3***	*DPYD*
*DRD1*	***DTL***	***DUSP10***	***EFNA5***	*ETV3*	***FAM83B***	***FBXL17***	*FBOX4*	*FOXR2 * ^2^	***GHR***
*GLYATL3*	***GNAI1***	***GNAT3***	*GPR158*	***GRIA1***	*GSTT4*	*GSTZ1 * ^2^	*HECTD2*	*HIP1R*	***HNMT***
***HNRNPH2***	*HOXA2*	*HYOU1*	***IGFBP2***	*IGIP*	*KCNE4*	*KLF7*	*KLHDC9 * ^2^	*LOC519202*	*LOC540403*
*LCO613444*	*LOC782977*	*LOC790004*	*LRRC31*	*LRRK2*	***LUZP2***	*MAGEH1 * ^2^	*MALT1*	*MED27*	*MID1IP1*
***MIF***	*MIIP*	*MIR135A-2*	*MIR2902*	*MYO3A*	*NDUFV3*	*NECTIN4 * ^2^	***NELL2***	*NTNG2*	*NUDCD1*
***PARVA***	*PCDH7*	*PHACTR1*	*PLOD2*	*PON1*	*PON3*	*POU3F3*	***PPIL4***	*PPM1A*	*PPP1R3C*
***PRIM2***	*PTBP2*	*PTTG1*	*RAB23*	*RAMP1*	*RFX7*	*RPL37A*	***RSAD2***	*SAMD7*	*SATB1*
*SKAP2*	*SLC1A4*	***SLC2A13***	*SLC16A3*	***SLC30A9***	*SLC37A4*	*SPDYC*	*STPG2*	*STUM*	***SYNCRIP***
*SYVN1*	*TBC1D7*	***TBL1XR1***	***TEAD1***	*TEF*	*TEX9*	*TFAP2A*	*THSD7A*	*THSD7B*	*TIFAB*
*TINAG*	*TMED8 * ^2^	***TMEM33***	*TMEM106B*	***TMEM117***	***TMPO***	*TOB2*	***TOX***	*TRHDE * ^2,3^	*TRHR*
*TSNAX*	*TTLL7*	*USP24*	*WDR4*	*ZNF365*					

^1^ Italic indicates gene names, red text indicates cancer genes and bold text indicates “IS-Clustered genes”. ^2^ Boxes text indicates genes related to multiple IS. ^3^ *CLSTN2* and *TRHDE* were related; both IS located in introns and intergenic regions.

**Table 2:** Gene ontology analysis of “IS-Related gene” for biological processes.

Term ^1^	Gene ^2^	*p*-Value
homophilic cell adhesion via plasma membrane adhesion molecules	*CDH10*, *CDH19*, *CDH7*, ***CLSTN2***, *NECTIN4*, ***PTPRM***, *PCDH7*	0.00344
nervous system development	***EPHB1***, *LDB2*, ***SMARCA4***, *ENFA5*, ***NAV2***, *NAV3*, ***PTEN***, *TFAP2A*	0.00432
positive regulation of receptor signaling pathway via JAK-STAT	***CAMK2A***, ***ERBB4***, ***GHR***, ***IL10***	0.00843
cell–cell adhesion mediated by cadherin	*FER*, *CDH10*, *CDH19*, *CDH7*	0.00843
modulation of chemical synaptic transmission	*DLGAP1*, ***GRIA1***, ***GRID2***, *NTNG2*, ***RTN4***	0.0104
regulation of focal adhesion assembly	***EFNA5***, *GPM6B*, ***VCL***	0.011
protein polyubiquitination	***FBXL17***, *FBXO4*, *HECTD2*, ***DTL***, ***RNF144A***, *UBE3D*	0.0173
regulation of neuron migration	***CAMK2A***, *NTNG2*, *PHACTR1*	0.0175
carboxylic acid catabolic process	*PON1*, *PON3*	0.0214
protein dephosphorylation	*RNGTT*, ***DUSP10***, ***PTEN***, *PPM1A*, ***PTPRM***	0.0285
positive regulation of transcription by RNA polymerase II	*LDB2*, *POU3F3*, ***SMARCA4***, ***TBL1XR1***, ***TEAD1***, *TEF*, *CCDC32*, ***HDAC4***, *HOXA2*, ***IL10***, *RFX7*, ***SLC30A9***, ***TOX***, *TFAP2A*, *ZNF407*	0.0298
cell adhesion	***EPHB1***, *FER*, ***ACAN***, *CNTNAP2*, *NECTIN4*, ***PARVA***, *PCDH7*, *TINAG*, ***VCL***	0.0308
actin cytoskeleton organization	*FER*, *ARHGEF10*, *MKLN1*, ***PARVA***, *THSD7A*, *THSD7B*	0.0309
regulation of neuron projection development	***GRID2***, *NTNG2*, *ZNF365*	0.0316
negative regulation of mast cell activation involved in immune response	*FER*, ***ENPP3***	0.0319
regulation of branching morphogenesis of a nerve	*LRRK2*, ***RTN4***	0.0319
lactone catabolic process	*PON1*, *PON3*	0.0319
synaptic membrane adhesion	*CDH10*, ***EFNA5***, *NTNG2*	0.0362
chemical synaptic transmission	*CARTPT*, ***CHRM1***, *EXOC4*, ***GABRB1***, ***GRM5***	0.0418
dendrite arborization	*PHACTR1*, *ZNF365*	0.0423
Neurogenesis	*CDK5RAP2*, ***ERBB4***, ***NAV2***, *NAV3*	0.0426

^1^ Underlined text indicates GO terms processes potentially involved in tumorigenesis. ^2^ Red text indicates cancer genes and bold text indicates “IS-Clustered genes”.

**Table 3:** Gene ontology analysis of “IS-Related gene” for KEGG pathway.

Term	Gene ^1^	*p*-Value
Dopaminergic synapse	***GNAI1***, ***ATF2***, ***CAMK2A***, ***CAMK2D***, *DRD1*, ***GRIA1***, ***PPP2R2B***	0.00394
Amphetamine addiction	***ATF2***, ***CAMK2A***, ***CAMK2D***, *DRD1*, ***GRIA1***	0.00708
Axon guidance	***EPHB1***, ***GNAI1***, ***CAMK2A***, ***CAMK2D***, *DPYSL5*, ***EFNA5***, *NTNG2*	0.0151
Pyrimidine metabolism	***CMPK2***, *DPYD*, ***ENPP3***, ***RRM1***	0.0274
Cholinergic synapse	***GNAI1***, ***CAMK2A***, ***CAMK2D***, ***CHRM1***, *KCNQ5*	0.0386
Long-term potentiation	***CAMK2A***, ***CAMK2D***, ***GRIA1***, ***GRM5***	0.0424

^1^ Bold text indicates “Clustered genes”.

**Table 4:** Gene ontology analysis of “IS-Clustered genes” for biological processes.

Term ^1^	Gene ^2^	*p*-Value
positive regulation of receptor signaling pathway via JAK-STAT	*CAMK2A*, *ERBB4*, *GHR*, *IL10*	0.000479
modulation of chemical synaptic transmission	*DLGAP1*, *GRIA1*, *GRID2*, *RTN4*	0.0037
signal transduction	*ADGRL2*, *CAMK2A*, *CAMK2D*, *CHN1*, *DUSP10*, *FAM83B*, *GABRB1*, *MYO10*, *PTPRM*	0.00394
nervous system development	*EPHB1*, *SMARCA4*, *EFNA5*, *NAV2*, *PTEN*	0.00639
ephrin receptor signaling pathway	*EPHB1*, *CHN1*, *EFNA5*	0.00785
positive regulation of synapse assembly	*CLSTN2*, *EFNA5*, *GRID2*	0.0135
positive regulation of miRNA transcription	*SMARCA4*, *TEAD1*, *IL10*	0.0158
NK T cell differentiation	*ITK*, *ATF2*	0.0195
protein phosphorylation	*EPHB1*, *ITK*, *CAMK2A*, *CAMK2D*, *ERBB4*, *SGK1*	0.0235
positive regulation of toll-like receptor 9 signaling pathway	*RSAD2*, *RTN4*	0.0271
monoatomic ion transport	*GABRB1*, *GRIA1*, *GRID2*	0.0277
cellular response to forskolin	*GNAI1*, *EFNA5*	0.0461
regulation of neuronal synaptic plasticity	*CAMK2A*, *CAMK2D*	0.0498

^1^ Underlined text indicates GO terms processes potentially involved in tumorigenesis. ^2^ Red text indicates cancer genes.

**Table 5:** Gene ontology analysis of “IS-Clustered genes” for KEGG pathway.

Term^1^	Gene ^2^	*p*-Value
Dopaminergic synapse	*GNAI1*, *ATF2*, *CAMK2A*, *CAMK2D*, *GRIA1*, *PPP2R2B*	0.000622
PI3K-Akt signaling pathway	*ATF2*, *CHRM1*, *EFNA5*, *ERBB4*, *GHR*, *PTEN*, *PPP2R2B*, *SGK1*	0.00356
Amphetamine addiction	*ATF2*, *CAMK2A*, *CAMK2D*, *GRIA1*	0.00522
Long-term potentiation	*CAMK2A*, *CAMK2D*, *GRIA1*, *GRM5*	0.00543
Adrenergic signaling in cardiomyocytes	*GNAI1*, *ATF2*, *CAMK2A*, *CAMK2D*, *PPP2R2B*	0.00852
cAMP signaling pathway	*GNAI1*, *TIAM1*, *CAMK2A*, *CAMK2D*, *CHRM1*, *GRIA1*	0.00888
Nucleotide metabolism	*ADK*, *CMPK2*, *ENPP3*, *RRM1*	0.0102
Axon guidance	*EPHB1*, *GNAI1*, *CAMK2A*, *CAMK2D*, *EFNA5*	0.0135
Circadian entrainment	*GNAI1*, *CAMK2A*, *CAMK2D*, *GRIA1*	0.0148
Proteoglycans in cancer	*TIAM1*, *CAMK2A*, *CAMK2D*, *ERBB4*, *MRAS*	0.0204
Cholinergic synapse	*GNAI1*, *CAMK2A*, *CAMK2D*, *CHRM1*	0.0209
Glutamatergic synapse	*DLGAP1*, *GNAI1*, *GRIA1*, *GRM5*	0.0209
Pyrimidine metabolism	*CMPK2*, *ENPP3*, *RRM1*	0.0365
Long-term depression	*GNAI1*, *GRIA1*, *GRID2*	0.0412
Retrograde endocannabinoid signaling	*GNAI1*, *GABRB1*, *GRIA1*, *GRM5*	0.044
Calcium signaling pathway	*CAMK2A*, *CAMK2D*, *CHRM1*, *ERBB4*, *GRM5*	0.0455
Cushing syndrome	*GNAI1*, *ATF2*, *CAMK2A*, *CAMK2D*	0.0468

^1^ Underlined text indicates KEGG pathway processes potentially involved in tumorigenesis. ^2^ Red text indicates cancer genes and bold text indicates “IS-Clustered genes”.

**Table 6:** Previously reported integration site-associated genes.

Gene ^1^	Reference
***ADRB2***, ***AGTR1***, *APOH*, ***BGLAP***, *BSP1*, *CD99*, *CHGB*, *FAM83D*, ***MTOR***	[28]
*ARHGEF4*, ***BRCC3***, *CDX1*, ***CGGBP1***, ***COL2A1***, ***DDX10***, *ELF2*, *FAM168B*, *FOXR2*, *GAS6*, ***HUWE1***, ***ICA1***, ***IRS2***, *KLHL14*, ***KPNA3***, ***KSR1***, *LHPP*, ***MSH2***, *MTCP1*, ***MYCBP2***, *N4BP2*, ***NAPG***, ***NF1***, *OSBPL8*, ***PDGFRB***, *PPP1R12C*, *PRPSAP2*, ***RAPGEF6***, ***RASA3***, ***RRAGB***, *SCAF8*, *SEPT11*, *SNIIP1*, *SPOCK1*, ***STARD7***, ***TCF4***, *TMEM67*, ***TNKS***, *UBASH3B*, ***UBE2D3***, ***WDR82***	[30]
***ANK3***, *FAM135B*, *FAM92A*	[32]
***CCDC71***, ***CITED4***, ***CTPS1***, ***LMBRD2***, ***NAMPT***, ***PGPEP1***, *SCFD2*, ***SKP2***	[33]
***PRTN4IP1***, ***ATG5*****, *RPTOR***	[34]
***CHEK2***	[35]

^1^ Bold text indicates gene incorporated “common EBL-related cluster” and red text indicates “ IS-Related genes” identified in the present study.

**Table 7:** Previously reported EBL-related genes.

Gene ^1^	References
***TP53***	[17,18,19,20,21]
***TP53***, ***KMT2D***, ***CREBBP***, ***KRAS***, ***PTEN***, ***NOTCH1***, ***MYD88***, ***CARD11***	[25]
***PRMT5***	[26]
***EXO1*** **, *MSH2***	[27]

^1^ Bold text indicates gene incorporated “common EBL-related cluster” and red text indicates “IS-Related genes” identified in the present study.

**Table 8:** Gene ontology analysis of “common EBL-related cluster” for biological processes.

Term ^1^	Gene ^2^	*p*-Value
**positive regulation of receptor signaling pathway via JAK-STAT**	***CAMK2A***, ***ERBB4***, ***GHR***, ***IL10***, *NOTCH1*	0.0000968
positive regulation of transcription by RNA polymerase II	*CREBBP*, *MYD88*, ***SMARCA4***, ***TBL1XR1***, ***TEAD1***, *ADRB2*, ***HDAC4***, ***IL10***, *KMT2D*, *NAMPT*, ***SLC30A9***, *TNKS*, ***TOX***, *TXF4*, *TP53*	0.000198
Ras protein signal transduction	*KRAS*, *RAPGEF6*, *KSR1*, ***MRAS***, *TP53*	0.000835
**protein phosphorylation**	***EPHB1***, ***ITK***, ***CAMK2A***, ***CAMK2D***, *CHEK2*, ***ERBB4***, *KSR1*, *MTOR*, *PDGFRB*, ***SGK1***	0.00111
negative regulation of autophagy	*RRAGB*, ***IL10***, *MTOR*, *RPTOR*	0.00179
**positive regulation of miRNA transcription**	***SMARCA4***, ***TEAD1***, ***IL10***, *TP53*	0.00345
TORC1 signaling	*CARD11* , *MTOR*, *RPTOR*	0.00378
cellular response to leucine starvation	*RRAGB*, ***ATF2***, *MTOR*	0.00378
mismatch repair	*EXO1*, *MSH2*, ***MSH3***	0.00544
Rac protein signal transduction	***FARP1***, *KRAS*, ***TIAM1***	0.00605
**nervous system development**	***EPHB1***, ***SMARCA4***, ***EFNA5***, ***NAV2***, ***PTEN***, *RTN4IP1*	0.00629
circadian regulation of gene expression	*HUWE1*, *MYCBP2*, *NAMPT*, *PRMT5*	0.00639
**signal transduction**	*KRAS*, ***ADGRL2***, *ANK3*, ***CAMK2A***, ***CAMK2D***, ***CHN1***, ***DUSP10***, ***FAM83B***, ***GABRB1***, ***MYO10***, ***PTPRM***	0.00719
positive regulation of peptidyl-tyrosine phosphorylation	***EFNA5***, ***GHR***, *MTOR*, *TP53*	0.0073
positive regulation of calcium-mediated signaling	***CA8***, ***GRM5***, *PDGFRB*	0.00957
protein polyubiquitination	***FBXL17***, *HUWE1*, ***DTL***, ***RNF144A***, *TNKS*	0.0109
oligodendrocyte differentiation	***DUSP10***, *MTOR*, *NOTCH1*	0.0112
intrinsic apoptotic signaling pathway in response to hypoxia	***ATF2***, *TP53*	0.0123
positive regulation of phosphatidylinositol 3-kinase/protein kinase B signal transduction	***ERBB4***, *IRS2*, *PDGFRB*, ***RTN4***, *TCF4*	0.0129
**modulation of chemical synaptic transmission**	***DLGAP1***, ***GRIA1***, ***GRID2***, ***RTN4***	0.013
outflow tract morphogenesis	***SEC24B***, ***ATF2***, *NOTCH1*	0.0157
positive regulation of G1/S transition of mitotic cell cycle	***ANKRD17***, *RPTOR*, *RRM1*	0.0167
anterior head development	*DDX10*, *COL2A1*	0.0184
**ephrin receptor signaling pathway**	***EPHB1***, ***CHN1***, ***EFNA5***	0.0187
glucose homeostasis	***CSMD1***, *BGLAP*, ***PRCP***, *TCF4*	0.02
positive regulation of epithelial to mesenchymal transition	*MTOR*, *NOTCH1*, *TCF4*	0.0208
protein import into nucleus	***ATF2***, *KPNA3*, *NOTCH1*, *TP53*	0.0223
Cognition	*BGLAP*, ***CHRM1***, *NF1*	0.0231
maintenance of DNA repeat elements	***MSH3***, *TCF4*	0.0245
**NK T cell differentiation**	***ITK***, ***ATF2***	0.0305
negative regulation of glial cell proliferation	*NOTCH1*, *TP53*	0.0305
regulation of blood vessel endothelial cell migration	*NF1*, *PRCP*	0.0305
**positive regulation of synapse assembly**	***CLSTN2***, ***EFNA5***, ***GRID2***	0.0316
mitotic recombination	*MSH2*, ***MSH3***	0.0365
regulation of somitogenesis	***DMRT2***, *NOTCH1*	0.0365
cellular response to follicle-stimulating hormone stimulus	***EFNA5***, *NOTCH1*	0.0365
DNA-templated transcription termination	*WDR82*, *PRMT5*	0.0365
**positive regulation of toll-like receptor 9 signaling pathway**	***RSAD2***, ***RTN4***	0.0425
coronary artery morphogenesis	***SEC24B***, *NOTCH1*	0.0425
skeletal system development	***ACAN***, *COL2A1*, ***HDAC4***	0.047
cardiac muscle cell apoptotic process	*ATG5*, *TP53*	0.0484
positive regulation of transcription of nucleolar large rRNA by RNA polymerase I	***SMARCA4***, *MTOR*	0.0484
thymocyte apoptotic process	*CHEK2*, *TP53*	0.0484

^1^ Bold text indicates common terms with “IS-Clustered genes”. ^2^ Red text indicates cancer genes and bold text indicates “IS-Clustered genes”.

**Table 9:** Gene ontology analysis of “common EBL-related cluster” for KEGG pathway.

Term ^1^	Gene ^2^	*p*-Value
**PI3K-Akt signaling pathway**	*KRAS*, ***ATF2***, ***CHRM1***, *COL2A1*, ***EFNA5***, ***ERBB4***, ***GHR***, *MTOR*, ***PTEN***, *PDGFRB*, ***PPP2R2B***, *RPTOR*, ***SGK1***, *TP53*	0.0000292
Glioma	*KRAS*, ***CAMK2A***, ***CAMK2D***, *MTOR*, ***PTEN***, *PDGFRB*, *TP53*	0.0000554
Longevity regulating pathway	*KRAS*, ***ATF2***, *ATG5*, *IRS2*, *MTOR*, *RPTOR*, *TP53*	0.000124
MicroRNAs in cancer	*CREBBP*, *KRAS*, ***EFNA5***, ***HDAC4***, *IRS2*, *MTOR*, *NOTCH1*, ***PTEN***, *PDGFRB*, *RPTOR*, *TP53*	0.000227
**Long-term potentiation**	*CREBBP*, * KRAS*, ***CAMK2A***, ***CAMK2D***, ***GRIA1***, ***GRM5***	0.000338
**Proteoglycans in cancer**	*KRAS*, ***TIAM1***, *ANK3*, ***CAMK2A***, ***CAMK2D***, ***ERBB4***, *MTOR*, ***MRAS***, *TP53*	0.000385
Growth hormone synthesis, secretion and action	*CREBBP*, ***GNAI1***, *KRAS*, ***ATF2***, ***GHR***, *IRS2*, *MTOR*	0.00059
Autophagy—animal	*KRAS*, *RRAGB*, *ATG5*, *IRS2*, *MTOR*, ***MRAS***, *PTEN*, *RPTOR*	0.000731
Pathways in cancer	*CREBBP*, ***GNAI1***, *KRAS*, *SKP2*, *AGTR1*, ***CAMK2A***, ***CAMK2D***, *MTOR*, *MSH2*, ***MSH3***, *NOTCH1*, *PTEN*, *PDGFRB*, *TP53*	0.000926
FoxO signaling pathway	*CREBBP*, *KRAS*, *SKP2*, *IRS2*, ***IL10***, ***PTEN***, ***SGK1***	0.00109
MAPK signaling pathway	*KRAS*, *MYD88*, ***ATF2***, ***DUSP10***, ***EFNA5***, ***ERBB4***, ***MRAS***, *NF1*, *PDGFRB*, *TP53*	0.00111
Prostate cancer	*CREBBP*, *KRAS*, *MTOR*, ***PTEN***, *PDGFRB*, *TP53*	0.00164
Longevity regulating pathway—multiple species	*KRAS*, *ATG5*, *IRS2*, *MTOR*, *RPTOR*	0.00189
**Adrenergic signaling in cardiomyocytes**	***GNAI1***, ***ATF2***, *ADRB2*, *AGTR1*, ***CAMK2A***, ***CAMK2D***, ***PPP2R2B***	0.00244
Central carbon metabolism in cancer	*KRAS*, *MTOR*, ***PTEN***, *PDGFRB*, *TP53*	0.00297
mTOR signaling pathway	*KRAS*, *RRAGB*, *SKP2*, *MTOR*, ***PTEN***, *RPTOR*, ***SGK1***	0.00303
Ras signaling pathway	*KRAS*, *RASA3*, ***TIAM1***, ***EFNA5***, *KSR1*, ***MRAS***, *NF1*, *PDGFRB*	0.0055
EGFR tyrosine kinase inhibitor resistance	*KRAS*, *MTOR*, *NF1*, ***PTEN***, *PDGFRB*	0.00551
**cAMP signaling pathway**	*CREBBP*, ***GNAI1***, ***TIAM1***, *ADRB2*, ***CAMK2A***, ***CAMK2D***, ***CHRM1***, ***GRIA1***	0.00599
**Dopaminergic synapse**	***GNAI1***, ***ATF2***, ***CAMK2A***, ***CAMK2D***, ***GRIA1***, ***PPP2R2B***	0.00645
ErbB signaling pathway	*KRAS*, ***CAMK2A***, ***CAMK2D***, ***ERBB4***, *MTOR*	0.00652
**Nucleotide metabolism**	*CTPS1*, ***ADK***, ***CMPK2***, ***ENPP3***, ***RRM1***	0.00707
**Calcium signaling pathway**	*ADRB2*, *AGTR1*, ***CAMK2A***, ***CAMK2D***, ***CHRM1***, ***ERBB4***, ***GRM5***, *PDGFRB*	0.00784
Apelin signaling pathway	***GNAI1***, *KRAS*, *AGTR1*, ***HDAC4***, *MTOR*, ***MRAS***	0.00794
Colorectal cancer	*KRAS*, *MTOR*, *MSH2*, ***MSH3***, *TP53*	0.00825
Phospholipase D signaling pathway	*KRAS*, *AGTR1*, ***GRM5***, *MTOR*, ***MRAS***, *PDGFRB*	0.0107
Rap1 signaling pathway	***GNAI1***, *KRAS*, *RAPGEF6*, ***TIAM1***, ***EFNA5***, ***MRAS***, *PDGFRB*	0.011
Human papillomavirus infection	*CREBBP*, *KRAS*, *COL2A1*, *MTOR*, *NOTCH1*, ***PTEN***, *PDGFRB*, ***PPP2R2B***, *TP53*	0.0114
**Cushing syndrome**	***GNAI1***, ***ATF2***, *AGTR1*, ***CAMK2A***, ***CAMK2D***, *KMT2D*	0.0119
Melanogenesis	*CREBBP*, ***GNAI1***, *KRAS*, ***CAMK2A***, ***CAMK2D***	0.0122
Cellular senescence	*KRAS*, *CHEK2*, *MTOR*, ***MRAS***, ***PTEN***, *TP53*	0.0138
**Pyrimidine metabolism**	*CTSP1*, ***CMPK2***, ***ENPP3***, ***RRM1***	0.0144
Mismatch repair	*EXO1*, *MSH2*, ***MSH3***	0.0167
**Long-term depression**	***GNAI1***, *KRAS*, ***GRIA1***, ***GRID2***	0.0172
**Cholinergic synapse**	***GNAI1***, *KRAS*, ***CAMK2A***, ***CAMK2D***, ***CHRM1***	0.0176
**Axon guidance**	***EPHB1***, ***GNAI1***, *KRAS*, ***CAMK2A***, ***CAMK2D***, ***EFNA5***	0.0203
Thyroid hormone signaling pathway	*CREBBP*, *KRAS*, *MTOR*, *NOTCH1*, *TP53*	0.0213
**Amphetamine addiction**	***ATF2***, ***CAMK2A***, ***CAMK2D***, ***GRIA1***	0.0219
Sphingolipid signaling pathway	***GNAI1***, *KRAS*, ***PTEN***, ***PPP2R2B***, *TP53*	0.0231
Neurotrophin signaling pathway	*KRAS*, ***ARHGDIA***, ***CAMK2A***, ***CAMK2D***, *TP53*	0.0231
T cell receptor signaling pathway	***ITK***, * KRAS*, *CARD11*, ***IL10***, ***PPP2R2B***	0.0249
Melanoma	*KRAS*, ***PTEN***, *PDGFRB*, *TP53*	0.0262
Autophagy—other	*ATG5*, *MTOR*, *RPTOR*	0.033
Tuberculosis	*CREBBP*, *MYD88*, ***CAMK2A***, ***CAMK2D***, ***IL10***, *KSR1*	0.0355
Chemical carcinogenesis—receptor activation	***GNAI1***, *KRAS*, ***ATF2***, *ADRB2*, *KPNA3*, *MTOR*	0.0407
Breast cancer	*KRAS*, * MTOR*, *NOTCH1*, ***PTEN***, *TP53*	0.042
Gap junction	***GNAI1***, *KRAS*, ***GRM5***, *PDGFRB*	0.0456
Endocrine resistance	*KRAS*, * MTOR*, *NOTCH1*, *TP53*	0.0493
PD-L1 expression and PD-1 checkpoint pathway in cancer	*KRAS*, *MYD88*, *MTOR*, ***PTEN***	0.0493

^1^ Bold text indicates common terms with “IS-Clustered genes” and underlined text indicates terms directly involved in cancer. ^2^ Red text indicates cancer genes and bold text indicates “IS-Clustered genes”.

## Data Availability

The original contributions presented in the study are included in the article; further inquiries can be directed to the corresponding author.

## Abbreviations

The following abbreviations are used in this manuscript:

BLV	Bovine leukemia virus
EBL	Enzootic bovine leucosis
CHEK2	checkpoint kinase 2
DAVID	Database for Annotation, Visualization, and Integrated Discovery
DNA	DNA repeat element
GO	Gene ontology
HTLV-1	Human T-cell leukemia viruses type 1
IS(s)	Integration site(s)
LINE	Long interspersed nuclear elements
LTR	Long terminal repeats
SINE	Short interspersed nuclear elements
